# Vitacrystallography: Structural Biomarkers of Breast Cancer Obtained by X-ray Scattering

**DOI:** 10.3390/cancers16142499

**Published:** 2024-07-09

**Authors:** Sergey Denisov, Benjamin Blinchevsky, Jonathan Friedman, Barbara Gerbelli, Ash Ajeer, Lois Adams, Charlene Greenwood, Keith Rogers, Lev Mourokh, Pavel Lazarev

**Affiliations:** 1 Matur UK Ltd., 5 New Street Square, London EC4A 3TW, UK; sdenisov@matur.co.uk (S.D.); bblinchevsky@matur.co.uk (B.B.); plazarev@matur.co.uk (P.L.); 2 Institut de Chimie Physique, UMR8000, CNRS, Université Paris-Saclay, Bât. 349, 91405 Orsay, France; 3 EosDx, Inc., 1455 Adams Drive, Menlo Park, CA 94025, USA; jonbenfri@gmail.com (J.F.); c.e.greenwood@keele.ac.uk (C.G.); k.d.rogers@cranfield.ac.uk (K.R.); 4 Physics Department, Queens College, City University of New York, 65-30 Kissena Blvd, Flushing, NY 11367, USA; 5 School of Chemical and Physical Sciences, Keele University, Keele ST5 5BG, UK; barbara.gerbelli@diamond.ac.uk (B.G.); a.ajeer@keele.ac.uk (A.A.); l.e.m.adams@keele.ac.uk (L.A.); 6 Shrivenham Campus, Cranfield University, Swindon SN6 8LA, UK

**Keywords:** structural biomarkers, X-ray scattering, extracellular matrix, cancer detection, machine learning

## Abstract

**Simple Summary:**

Breast cancer ranks as the most prevalent cancer among women. Current screening includes regular mammography and subsequent biopsy if the mammography results are abnormal. These procedures are costly and uncomfortable. We propose an alternative non-invasive method based on X-ray scattering. Using a machine learning approach, we have examined almost 3000 measurements of cancerous and non-cancerous samples belonging to 110 patients and shown excellent results on cancer/non-cancer separation. This can lead to patient-friendly, fast, and economical solutions for breast cancer screening to complement mammography and reduce biopsy. It should be emphasized that this approach can be readily extended to other types of cancer and even other diseases.

**Abstract:**

With breast cancer being one of the most widespread causes of death for women, there is an unmet need for its early detection. For this purpose, we propose a non-invasive approach based on X-ray scattering. We measured samples from 107 unique patients provided by the Breast Cancer Now Tissue Biobank, with the total dataset containing 2958 entries. Two different sample-to-detector distances, 2 and 16 cm, were used to access various structural biomarkers at distinct ranges of momentum transfer values. The biomarkers related to lipid metabolism are consistent with those of previous studies. Machine learning analysis based on the Random Forest Classifier demonstrates excellent performance metrics for cancer/non-cancer binary decisions. The best sensitivity and specificity values are 80% and 92%, respectively, for the sample-to-detector distance of 2 cm and 86% and 83% for the sample-to-detector distance of 16 cm.

## 1. Introduction

Breast cancer is currently the leading type of new cancer cases and the second leading cause of cancer mortality for women. More than three hundred thousand new cases of breast cancer and more than forty thousand deaths are estimated for the US in 2024 [1]. While the mortality rate has slightly decreased over the last few years [1], the total number is still worryingly large. The decrease in mortality can be attributed to early diagnosis of breast cancer [2], and thus, the development of early-stage biomarkers is paramount. To address this, we propose monitoring the status of extracellular matrix components using X-ray scattering.

The extracellular matrix (ECM) [3,4] is the noncellular part of tissue with components synthesized mainly by cells. It provides mechanical support for cells and plays a significant role in maintaining tissue homeostasis. The ECM is constantly remodeled to adapt to various stresses [5], and this process generally becomes deregulated in the case of disease [6,7]. Specifically, notable changes in the structure of ECM molecules can indicate a tumor’s existence and progression and, correspondingly, serve as biomarker origins [8,9].

Since their discovery, X-rays have been widely used in medicine. However, early and current applications, such as fluorography, radioscopy, and mammography, are based on absorption processes, with X-ray scattering not explicitly exploited. Initially, X-ray crystallography was used to determine atom arrangements in crystalline solids. Later, the possibility of crystallizing biological molecules led to the development of biocrystallography, i.e., the revealing of the molecular content of complex structures; see [10,11] for historical overviews. However, the X-ray scattering approach can be applied to biological molecules or molecular complexes without crystallization, and, if there are periodic structures, they will manifest themselves in scattering patterns. In this, the periodicity, *d*, of the structure will appear as momentum transfer, *q* = 2π/*d*. While ECM components, such as fibrous proteins (collagens, elastins, fibronectins, and laminins) and glycoproteins, are not crystalline per se, their structural periodicity emerges during synthesis. To separate the studies of such objects from biocrystallography, we introduce the term vitacrystallography, which appears in the title of this paper.

Increasing numbers of reports use X-ray scattering to examine the physicochemical features of breast tissue ECM. Molecular imaging from X-ray scattering has been employed to examine collagen types derived in vivo from intralobular, functionally anomalous fibroblasts associated with invasive tumors. Initially, studies examined tertiary-level collagen structures, leading to new breast tumor microenvironment models [12,13]. Subsequently, multiple studies have examined X-ray scattering (coherent and incoherent) to probe the atomic scales of breast tissues. These have demonstrated high levels of imaging contrast based upon tissue type [14,15,16]. More recently, the physicochemical properties of ECM microcalcifications (small calcium phosphate deposits typically 10–100s μm in size) frequently associated with breast cancers, especially ductal carcinomas, have been reported. The chemical and microstructural characteristics of apatite (type II) microcalcifications act as immortalized biomarkers of the tissue environment when calcifications form, i.e., at the onset of pathological change. Thus, such features may provide a record of tissue changes from genesis to invasion. Atomic-scale X-ray scattering unlocks this information to indicate both intracellular and extracellular ionic concentrations [17,18,19,20,21] and other characteristics. This is relevant to, for example, elevated intracellular Na and Mg associated with mitogenesis. Such data would impact upon research into overtreatments as Mg is critical to cellular temporal benchmarking.

Another component that can be monitored in relation to cancer progression is lipids. The relationship between cancer and lipid metabolism was first reported in the mid-60s [22,23], with recent studies revisiting this relationship [24,25,26,27,28]. Cancer cells modify lipid metabolism, activating, desaturating, or elongating fatty acids. Moreover, de novo synthesized lipids can differ from those in circulation, affecting the lipid composition of tumors. These changes in lipid profile can be used to establish biomarkers. Currently, the primary analytic tool is mass spectroscopy [24,29], but lipids can be addressed directly using X-ray diffraction. For example, at least two diffraction maxima from breast tissue scattering are uniquely associated with lipid structures. One of them, the wide-angle maximum at *q* = 13.9 nm^−1^, corresponds to inter-fatty-acid molecular distances. It was shown [30,31,32] that for cancerous tissues, the intensity of this maximum reduces (often to <3σ_background_), while another peak at *q* = 20.2 nm^−1^ concomitantly increases in intensity. This maximum is attributed to the oxygen–oxygen distance in the tetrahedral structure of water [31]. Accordingly, it can be used as a structural biomarker. It should be noted that the values of *q* are off by 4π in [14,15,33], where it was reported that healthy adipose tissue exhibits a narrow peak at *q* = 1.1 nm^−1^, while for cancerous tissues, this peak appears shifted to *q* = 1.5 nm^−1^. When multiplied by 4π, it would give values similar to [30,31,32]. In small-angle scattering, the peak at *q* = 1.5 nm^−1^ has been attributed to the regular packing of triglyceride molecules and is a characteristic of healthy tissue [31,34,35]; moreover, it was shown [35] that this peak is absent in benign tissues. Another work [36] reported a peak at *q* = 1.725 nm^−1^ that appears primarily associated with malignant lesions.

The metastatic spread of breast cancer often involves bone as a secondary site. Although ‘bone homing’ mechanisms are poorly understood, some early indications of the role of local mineral environments are starting to emerge [37]. Many fundamental questions are associated with osteo-tumors that currently hinder the progression of early-stage diagnostics and effective therapies. For example, dormancy processes and mesenchymal–epithelial transitions have been difficult to characterize but are essential for understanding the natural history of metastatic cancers [38,39]. Dormancy and other components (progression) of the metastatic cascade are undoubtedly influenced by tumor microenvironments [40]. X-ray scatter imaging can critically provide information simultaneously on bone mass, architecture, and material ‘quality’ associated with microenvironments [41]. Further, detection of bone cancer at significantly earlier stages than currently possible with conventional radiology within animal models would be possible due to the enhanced contrast afforded by the technique. X-ray scattering has already been demonstrated to be capable of providing new biomarkers for osteoporosis and fracture prediction using atomic scale features (0.4–0.1 nm) averaged over ~mm^3^ mineral volumes [42,43]. Simultaneous architectural imaging can also be employed, using finite element modeling, to provide mechanical properties of the tissue and thus indicate the degradation of bone strength as cancer progresses.

In this paper, we report the analysis of data produced by measurements of X-ray scattering of breast tissues. We use two distinct sample-to-detector distances to address different *q*-ranges. The distance of 2 cm provides a *q*-range of up to 22.5 nm^−1^, while the distance of 16 cm magnifies small-angle scattering with a *q*-range of up to 4.5 nm^−1^. These two distances are chosen to examine both ranges associated with cancer-induced lipid transformations. We measure samples from 107 unique patients, obtaining 2958 distinctive scattering patterns. After azimuthal integration, these patterns are converted to dependencies of the scattered intensity on *q*. The averaged curves exhibit the previously obtained results, with a *q* = 1.5 nm^−1^ peak predominantly appearing in healthy tissues; suppression of the *q* = 13.9 nm^−1^ peak; and the appearance of a *q* = 20.2 nm^−1^ peak in cancerous samples. However, the total number of samples in our research significantly exceeds that of previous studies. It became possible for two reasons. First, many samples can be readily processed as we use a developed table-top diffractometer instead of a synchrotron. Second, a machine-learning technique has been employed for data analysis.

## 2. Materials and Methods

### 2.1. Experimental Design

#### 2.1.1. Breast Tissue Specimens

A total of 214 fresh-frozen core biopsy breast tissue specimens totaling 107 patients were utilized for this study and were obtained via the Breast Cancer Now Tissue Biobank. Two specimens per patient were provided for the study to assess heterogeneity. The donors were all female and ranged from 19 to 93 in age and included 60 patients diagnosed with cancer and 47 non-cancer patients. Ethical approval for the project was obtained locally via Keele University (NS-210096), and for the collection and use of specimens via the BCN tissue biobank (NRES Approval Number 21/EE/0072).

#### 2.1.2. Sample Preparation

**Figure 1:**
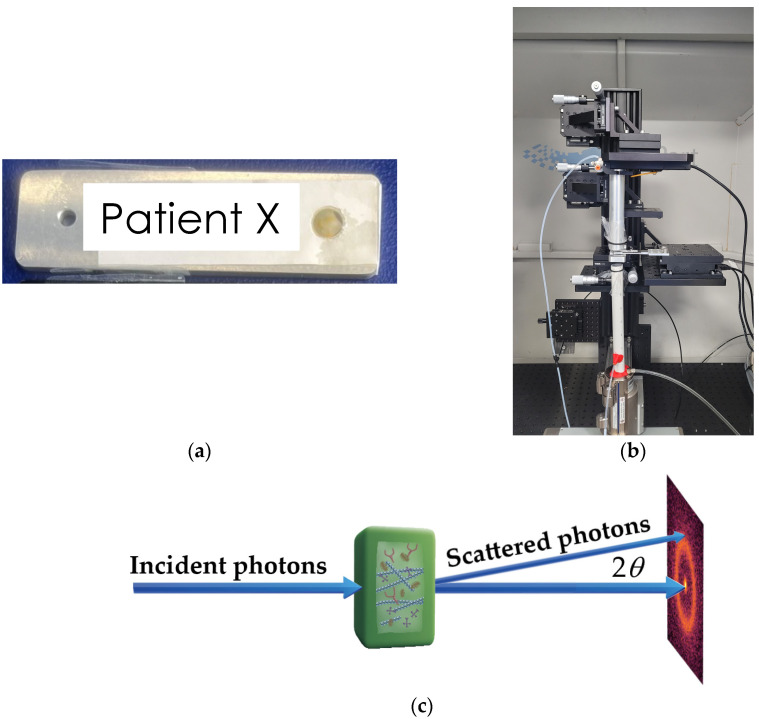
(**a**) Human breast tissue sealed within a 5 mm aperture bespoke sample holder; (**b**) EosDx X-ray diffractometer; (**c**) schematics of the experiment.

Each tissue specimen was placed into a bespoke aluminum sample holder of 2 mm thickness utilizing a SPEX^TM^ 6 µm thick mylar window film to seal and secure the tissue within a 5 mm aperture, as shown in Figure 1a. Crucially, the sample holders were designed to ensure the tissues remained hydrated throughout the analysis, which was validated by utilizing porcine tissue prior to scanning human tissue. To ensure consistency in sample-to-detector distances and measurements, the volume of tissue fully filled the sample holder. Silver behenate powder (Thermoscientific^®^ 045494.06, Waltham, MA, USA) was also scanned (utilizing the bespoke aluminum holders) to allow for accurate sample-to-detector distance measurements.

#### 2.1.3. X-ray Diffraction (XRD) Measurements

XRD analysis was carried out using a bespoke X-ray diffractometer (Figure 1b) engineered and built by EosDx, Inc. (Menlo Park, CA, USA), a US-based company developing X-ray scattering for medical diagnostics. The radiation produced by the Incoatec Microfocus Source copper source was collected with two multilayer curved mirror optics, resulting in a low-divergence monochromatic beam with a radiation wavelength *λ* = 0.154 nm. The two-dimensional detector was an MiniPix SN1442 Si 500 µm detector (ADVACAM, Prague, Czech Republic) with a 256 by 256 pixel array and a 55 by 55 m pixel size. Schematics of the experiment are shown in Figure 1c. The incident X-ray beam hits the sample containing various biomolecules and is scattered by the angle 2*θ*. The experimental data were stored as a 256 by 256 matrix of integers representing the photon counts. All the experiments were performed at room temperature (19 °C) under atmospheric pressure. To assess specimen heterogeneity, specimens were mapped with data collected from 10 individual spots on the sample, providing 10 diffractograms per specimen. Each diffractogram was collected for 3 min with a count time of 0.1 s. Background data were collected from an empty aluminum sample holder sealed utilizing mylar (as described above) and subtracted from sample data.

### 2.2. Data Analysis

The first step of data analysis was removing images from the database that were either empty or lacked a defined beam center, ensuring that only relevant data proceeded to the analysis stage. Following data cleaning, the center of each measured pattern was identified using the Pyfai module, and the precise sample-to-detector distance was determined using a calibration based on AgBH diffraction. The next step was azimuthal integration around the center to obtain radial profiles, effectively translating 2D image data into 1D dependence of the intensity of scattered X-rays on the distance from the center. Initially expressed in pixel units, the radial profiles were converted into momentum transfer *q* = (4π sin *θ*)/*λ*, where sin 2*θ* was calculated as the ratio of the distances from the pixel to the center and from the sample to the detector; see Figure 1c.

The dataset was clustered into two groups corresponding to the two different experimental sample-to-detector distances. The *q*-ranges were interpolated within each cluster to achieve a uniform length of 50 points, enhancing comparability, with a 4.5% cut from both sides of the *q*-range. This step involved the removal of outliers using the z-score technique to ensure the reliability of the interpolation process.

Prior to classification, the radial profiles underwent normalization and scaling. This step, executed with the scikit-learn library [44], involved adjusting the data within each cluster to have a standard distribution. The processed data from each cluster underwent a randomized separation (200 times) with respect to patients, with a train-test split of an approximately 60/40 ratio, forming not-overlapping subsets, i.e., training was performed using certain patients, and the test was accomplished with the other subset.

The machine learning algorithm from the scikit-learn library, Random Forest Classifier, was employed to classify the samples into cancerous and non-cancerous categories. This algorithm was chosen for its robustness and ability to handle complex patterns in data. The training phase involved adjusting the models to best fit the training data, while the testing phase assessed their performance in classifying unseen data. The number of trees was set to 100; the depth of trees was set to 10; and the learning rate of 0.01 was set for Random Forest. The learning was performed for all train-test subsets; 200 for each cluster.

We evaluated our model using sensitivity and specificity as our performance metrics. Sensitivity refers to the proportion of tumor samples that were accurately identified, while specificity relates to the accuracy in identifying control samples. To determine the most effective threshold for classification, we selected the one that brought our model’s performance closest to that of an ideal classifier, as indicated on the receiver operating characteristic (ROC) curve. The area under the ROC curve (AUC) is another critical measure, with values closer to one indicating superior performance.

## 3. Results

The samples were measured at two sample-to-detector distances. The descriptions of the collected datasets are shown in Table 1.

**Figure 2:**
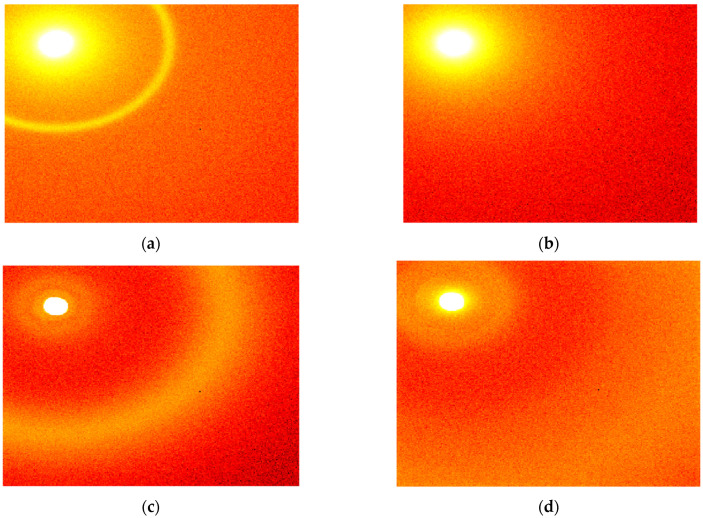
Typical X-ray scattering patterns obtained for (**a**) non-cancer samples measured at the sample-to-detector distance of 16 cm, (**b**) cancer samples measured at 16 cm, (**c**) non-cancer samples measured at 2 cm, and (**d**) cancer samples measured at 2 cm.

Typical results of the measurements are shown in Figure 2.

**Figure 3:**
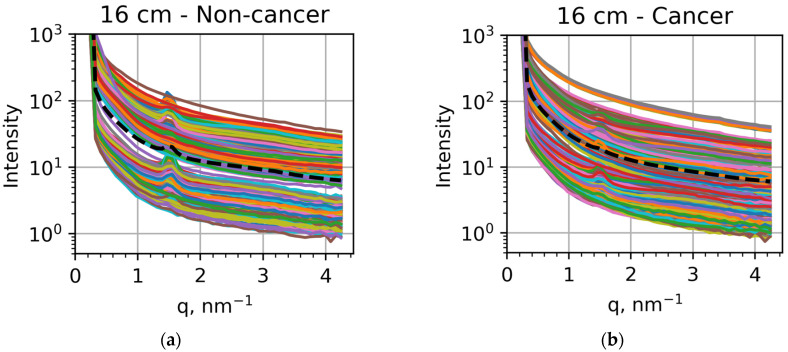

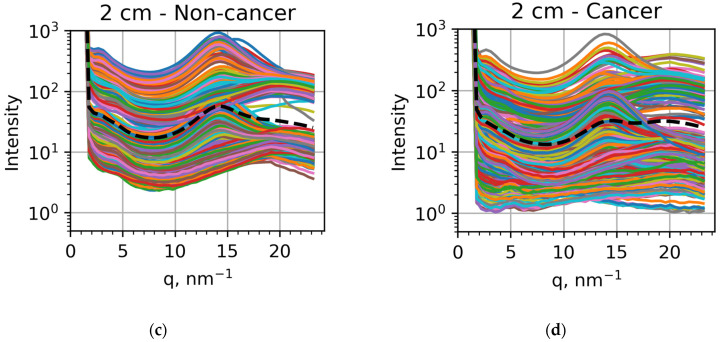
Dependencies of the scattered intensities on the transfer momentum obtained for (**a**) non-cancer samples measured at the sample-to-detector distance of 16 cm, (**b**) cancer samples measured at 16 cm, (**c**) non-cancer samples measured at 2 cm, and (**d**) cancer samples measured at 2 cm. The intensities for each specific sample are showed at different colors, with the averaged intensities shown as black dashed curves.

As shown in Figure 2, the scattering patterns are almost isotropic, and the azimuthal integration can be performed without losing information. As a result of this integration, the scattered intensity can be represented as a function of the distance to the center, which, in turn, can be rearranged in terms of the transfer momentum magnitude. The obtained dependencies of the intensity of the scattered X-rays on the transfer momentum are shown in Figure 3. The 2 cm cluster includes the region of 2.3–22.5 nm^−1^ of the transfer momentum magnitude, while the 16 cm cluster covers the 0.44–4.29 nm^−1^ range.

If we compare the averaged intensities, shown as black dashed curves, we can confirm the results of previous studies [37,38,39,40,41,42] related to lipid metabolism. For the 16 cm measurements, the peak at *q* = 1.5 nm^−1^ is very pronounced in non-cancer samples (Figure 3a) but much less visible in cancer samples (Figure 3b). For the 2 cm measurements, the peak at *q* = 14 nm^−1^ is clearly seen in non-cancer samples (Figure 3c) but less pronounced than the peak at *q* = 20 nm^−1^ in cancer samples (Figure 3d). These findings confirm previously obtained results relating these peak modifications to lipid metabolism [30,31,32,34,35]. However, the individual measurements can be significantly different from these general trends, as seen in all the panels of Figure 3. In particular, the 1.5 nm^−1^ peak appears in many cancer samples (Figure 3b). In contrast, the 20 nm^−1^ peak dominates the one at 14 nm^−1^ for many non-cancer samples (Figure 3c). Correspondingly, to reveal all the tendencies and facilitate the applications of this approach to actual diagnostics, we applied machine learning techniques.

**Figure 4:**
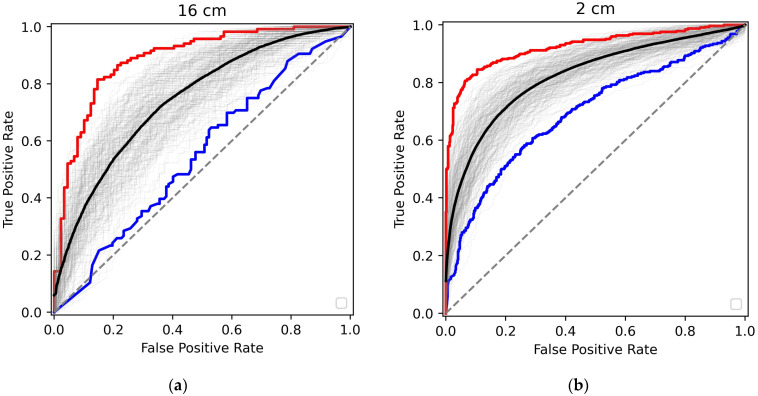
The ROC curves for various realizations of the training-test shuffles. (**a**) Measurements at the sample-to-detector distance of 16 cm and (**b**) at the sample-to-detector distance of 2 cm. The best (highest ROC AUC), worst (lowest ROC AUC), and averaged realizations are shown in red, blue, and black, respectively.

We trained the Random Forest Classifier procedure on 60% of measurements, using the remaining 40% for the test to determine the outcome metrics, with the same percentage of cancer patients in both clusters as in the total dataset. Several measurements of a single patient were kept in the same training or test clusters. Such training/test sequences were performed 200 times. The ROC curves for the test sets are shown in Figure 4 for both 16 cm and 2 cm measurements.

With each of the 200 realizations exhibited in grey, the best and the worst are shown in red and blue, respectively. The average curve is black. The corresponding performance metrics are presented in Table 2.

## 4. Discussion

It is evident from Figure 4 and Table 2 that the Random Forrest Classifier approach allows promising cancer/non-cancer binary diagnostics. For the best realization, the AUC ROC reached 94% for the 2 cm measurements and 87% for the 16 cm measurements. The metrics for the average realization are slightly worse but still very good. As illustrated in Table 2, the test specificity is demonstrated to possess credible metrics consistent with confirmatory diagnostic tests following initial positive screening. In such cases, high specificity becomes critical to ensure that patients identified as having cancer truly have the disease and to reduce the number of false positives and overtreatment. In general, the performance metrics are better for the 2 cm experiments. We believe this is due to the extremely small *q*-range, with significant contributions from the primary beam and small-angle amorphous scattering, which have more minor effects on data analysis in this case.

It should be emphasized that the most prominent features for both visual analyses based on Figure 3 and performance metrics obtained in machine learning are related to lipid metabolism. In the presence of a tumor, regular triglyceride packing is broken, suppressing the 1.5 nm^−1^ peak. Tumor cells also affect fatty acids, so the 13.9 nm^−1^ peak, corresponding to inter-fatty-acid molecular distances in healthy tissues, becomes less pronounced. It is crucial to include the other components of ECM in our analysis. However, the resolution on our diffractometer was too low to observe discrete collagen peaks at a low *q*, which is the direction of future improvements.

In the present paper, the measurements were performed on small samples obtained from biopsies. However, the same structural biomarkers determined in our studies would also appear in large samples and, eventually, in in vivo procedures. The developed table-top diffractometer should be modified for these future applications. Instead of the current Cu source, with a penetration depth of several mm, a Mo or Ag source will be used. They have much shorter wavelengths, leading to more negligible absorption and a penetration depth of several hundred mm. Moreover, the design of the diffractometer must be changed to accommodate in vivo measurements, and the current device is a prototype on the path to this ultimate goal. As biomarker determination is based on X-ray diffraction, not absorption, the received dose will be negligible. It should also be emphasized that at least small-sample studies are not limited to breast cancer. X-ray scattering measurements can determine structural biomarkers associated with other types of cancer and even other diseases.

## 5. Conclusions

In conclusion, we performed X-ray scattering measurements of breast tissue samples from 107 patients at two sample-to-detector distances of 2 and 16 cm. After azimuthal integration of the acquired patterns, we obtained the dependencies of scattered intensity on the momentum transfer magnitudes, exhibiting lipid structure features. The peaks associated with the regular packing of triglyceride molecules (at 1.5 nm^−1^) and with inter-fatty-acid molecular distances (at approximately 14 nm^−1^) were observed predominantly in non-cancer tissues, which confirms the results of previous studies, on average. To examine the feasibility of cancer detection on an individual level, we performed a statistical analysis based on the machine-learning Random Forest Classifier approach. We separated the total dataset into 60/40 training/test splits and repeated this separation 200 times, with the training and test splits not overlapping regarding patients. The obtained performance metrics vary from very good to excellent, with the sensitivity and specificity reaching 86% and 83%, respectively, for the best realization. We believe such results show great promise for future non-invasive technology for breast cancer detection.

## Figures and Tables

**Table 1:** Datasets for X-ray scattering data measured at two different distances of 2 and 16 cm.

Clusters	Diagnosis		
	Cancer	Non-Cancer	Total
Cluster 2 cm	825	1463	2288
Cluster 16 cm	306	364	670
	1131	1827	2958

**Table 2:** Random Forest performance metrics for 2 cm and 16 cm datasets.

Metrics Distances (cm)	AUC ROC		Sensitivity		Specificity	
	2	16	2	16	2	16
Random Forests (min)	64	58	56	78	73	36
Random Forests (max)	92	89	80	86	92	83
Random Forests (average)	78	73.5	68	82	82.5	59.5

## Data Availability

All produced data, including raw measurement data and the Jupiter notebooks for statistical analysis, will be made readily available by authors on request.
